# *Helicobacter pylori* infection in the stomach induces neuroinflammation: the potential roles of bacterial outer membrane vesicles in an animal model of Alzheimer’s disease

**DOI:** 10.1186/s41232-022-00224-8

**Published:** 2022-09-05

**Authors:** Ah-Mee Park, Ikuo Tsunoda

**Affiliations:** Department of Microbiology, Kindai University Faculty of Medicine, 377-2 Ohnohigashi, Osakasayama, Osaka, 589-8511 Japan

**Keywords:** *Helicobacter pylori*, Microglia, Neuroinflammation, Outer membrane vesicles, Mice, Alzheimer’s disease, Blood-brain barrier

## Abstract

*Helicobacter pylori* (HP) is a Gram-negative bacterium that colonizes the human stomach chronically. Colonization of HP in the gastric mucosa not only causes gastrointestinal diseases, but also is associated with extra-gastric diseases, such as idiopathic thrombocytopenic purpura and neurological diseases. Among neurological diseases, epidemiological studies have shown that HP infection increases the prevalence of Alzheimer’s disease (AD) and Parkinson’s disease (PD). Since HP does not invade the central nervous system (CNS), it has been considered that systemic immunological changes induced by HP infection may play pathogenic roles in AD and PD. Here, we investigated the effects of HP infection on the CNS in vivo and in vitro. In the CNS, chronically HP-infected mice had microglial activation without HP colonization, although systemic immunological changes were not observed. This led us to explore the possibility that HP-derived outer membrane vesicles (HP-OMVs) could cause neuroinflammation. OMVs are small, spherical bilayer vesicles (20–500 nm) released into the extracellular space from the outer membrane of Gram-negative bacteria; OMVs contain lipopolysaccharide, proteins, peptidoglycan, DNA, and RNA. OMVs have also been shown to activate both innate and acquired immune cells in vitro, and to disrupt the tight junctions of the gastric epithelium (“leaky gut”) as well as cross the blood-brain barrier in vivo. Thus, in theory, OMVs can activate immune responses in the remote organs, including the lymphoid organs and CNS, if only OMVs enter the systemic circulation. From the exosome fraction of sera from HP-infected mice, we detected HP-specific DNA, suggesting the presence of HP-OMVs. We also found that microglia incubated with HP-OMVs in vitro increased the cell proliferation, inflammatory cytokine production, and migration. On the other hand, HP-OMVs suppressed the cell proliferation of neuroblastoma in vitro. Lastly, we found that AD model mice infected with HP had amyloid plaques adjacent to activated microglia and astrocytes in vivo. Based on the literature review and our experimental data, we propose our working hypothesis that OMVs produced in chronic HP infection in the gut induce neuroinflammation in the CNS, explaining the higher prevalence of AD in HP-infected people.

## Introduction: *Helicobacter pylori* and gastric/extra-gastric diseases

**Fig. 1:**
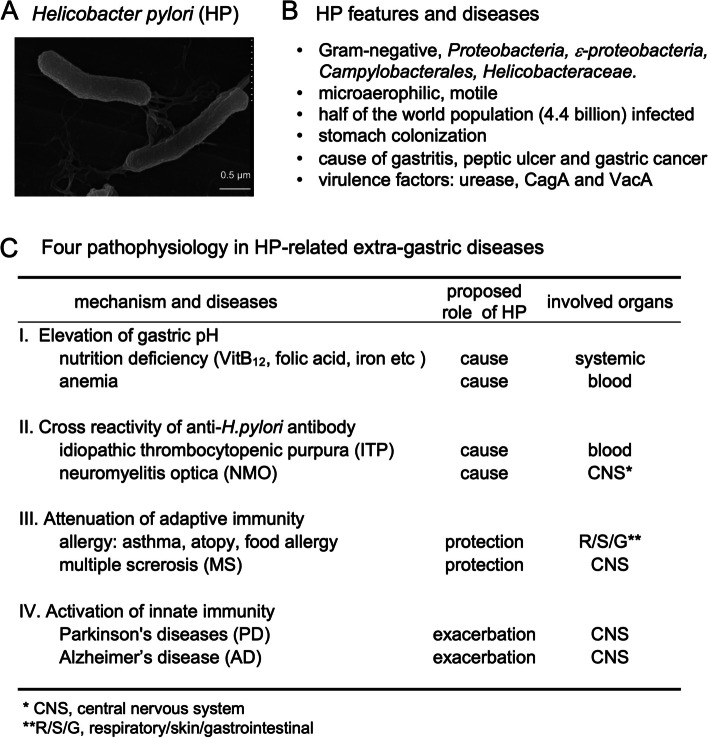
*Helicobacter pylori* (HP) and gastric/extra-gastric diseases. **A** Electron micrograph of HP, a spiral-shaped bacterium with multiple polar flagella at a single pole; the length is 2.5–4 μm and diameter is 0.3–0.5 μm. **B** HP features and diseases in the stomach. **C** HP-related extra-gastric diseases can be categorized into four groups based on the pathophysiology

*Helicobacter pylori* (HP) is a Gram-negative bacterium that belongs to the phylum *Proteobacteria,* class *ε-proteobacteria,* order *Campylobacterales,* family *Helicobacteraceae*, and genus *Helicobacter*; HP is a microaerophilic, spiral-shaped, highly mobile bacterium. The length and diameter of HP are 2.5–4 μm and 0.3–0.5 μm, respectively (Fig. 1A). HP colonizes the human stomach selectively and infects about 50% of the world’s population, approximately 4.4 billion individuals [1–3]. HP is divided into four major strains based on its core genome sequence: East Asia, Europe, Africa, and Amerind strains [4]. The HP colonization in the gastric mucosa causes peptic ulcers, chronic gastritis, gastric adenocarcinoma, and mucosa-associated lymphoid tissue (MALT) lymphoma (Fig. 1B); the major HP virulence factors are urease, cytotoxin-associated gene A (CagA), and vacuolating toxin A (VacA) [2]. Urease has two pathogenic roles by producing ammonia from urea: (1) ammonia neutralizes the gastric acid, which allows the colonization of HP that can grow only at neutral pH; and (2) ammonia itself damages the gastric epithelium. CagA alters intracellular signal transduction pathways that facilitate the malignant transformation of gastric epithelial cells [5]. Populations expressing a high incidence of gastric cancer are mostly identical to regions where East Asian type CagA is predominant [6]. VacA causes vacuolation and apoptosis of the epithelial cells, resulting in disrupting tight junctions of the epithelium (“leaky gut”), where the penetration of VacA into the lamina propria induces gastric inflammation [7, 8]. The “leaky gut” is the condition of increased intestinal permeability that occurs when tight junctions in the gut are damaged, such as by dysbiosis or chronic stress; the “leaky gut” can allow partially digested food and toxins to enter the bloodstream [9].

HP infection has also been associated with extra-gastric diseases, such as idiopathic thrombocytopenic purpura (ITP) and neurological diseases [10–13] (Fig. 1C). Although the precise mechanisms by which HP infection causes, exacerbates, or attenuates these diseases are unknown, the diseases can be categorized into four groups based on pathophysiology (Fig. 1C). One is due to elevated intragastric pH. Since HP infection elevates the gastric pH levels that result in poor absorption of various nutrients, including vitamin B_12_, folic acid, and iron, these nutrient deficiencies can cause various diseases, including anemia [14, 15].

On the other hand, the other three groups are related to immunological changes (Fig. 1C); three distinct immunopathogeneses have been linked to extra-gastric diseases in HP infection. First, antibodies against HP can cross-react with host antigens and damages host cells due to molecular mimicry between HP and human proteins. For example, ITP seems to be caused by molecular mimicry between HP-CagA and glycoprotein (GP) IIb/IIIa on the platelets [10]. The incidence of ITP is higher in patients with HP infection. In ITP, HP eradication has increased the platelet count and decreased circulating anti-GPIIb/IIIa antibody-producing B cells [10]. Neuromyelitis optica (NMO) is an inflammatory demyelinating disease in the central nervous system (CNS) caused by an autoantibody against aquaporin 4 (AQP4), a water channel in astrocytes. Molecular mimicry between HP-neutrophil-activating protein and AQP4 may play a role in disease exacerbation [16].

Second, HP infection has been shown to increase regulatory T cells (Tregs) that can suppress adaptive immune responses, resulting in modulation of extra-gastric diseases [17–19]. For example, the suppression of allergic immune responses by increased Tregs could explain the negative correlation between HP infection and allergic diseases such as asthma, atopy, and food allergy [17]. Since multiple sclerosis (MS), an inflammatory demyelinating disease in the CNS, has been associated with autoimmune T and B cell responses against CNS antigens, the lower incidence of MS in HP-infected patients may be due to the increase of Tregs [18, 19].

Third, HP infection may contribute to the pathogenesis of CNS diseases by activating innate immunity. The enhanced levels of pro-inflammatory cytokines, such as interleukin (IL)-1β, IL-6, and tumor necrosis factor (TNF)-α [20], as well as C-reactive protein (CRP) [21], have been detected in sera from HP-infected patients. Additionally, a higher incidence of HP infection has been reported in patients with Parkinson’s disease (PD) [13, 22] and Alzheimer’s disease (AD) [23, 24], compared with the controls. In both PD and AD, activation of brain resident innate cells, particularly microglia, is a common characteristic of CNS pathology. In a case-control study of PD, Bu et al. proposed that activating systemic innate immunity could contribute to CNS pathology; the authors speculated that HP-induced peripheral IL-1β and TNF-α might be delivered to the brain, leading to microglial activation in the CNS [25]. Although similar immunopathological changes may play pathogenic roles in AD, few experimental findings have been reported. Thus, we have investigated the roles of HP in neuroinflammatory diseases using in vivo and in vitro systems. In this article, we will introduce our experimental findings and propose our working hypothesis on HP infection and AD pathology.

### Microglial activation in the brain of HP-infected mice

Only a few animal studies have examined the relationship between neuroinflammation and infection with bacteria that belong to the genus *Helicobacter*. Gorlé et al. reported the induction of gastritis in mice infected with *Helicobacter suis*, which is known to colonize pigs’ stomachs [26]. One month after infection, the authors found microglial activation, which was accompanied with systemic inflammation, where increased plasma IL-1β levels were detected. On the other hand, there are no reports showing the relationship between HP (*H. pylori*) infection and CNS diseases.

**Fig. 2:**
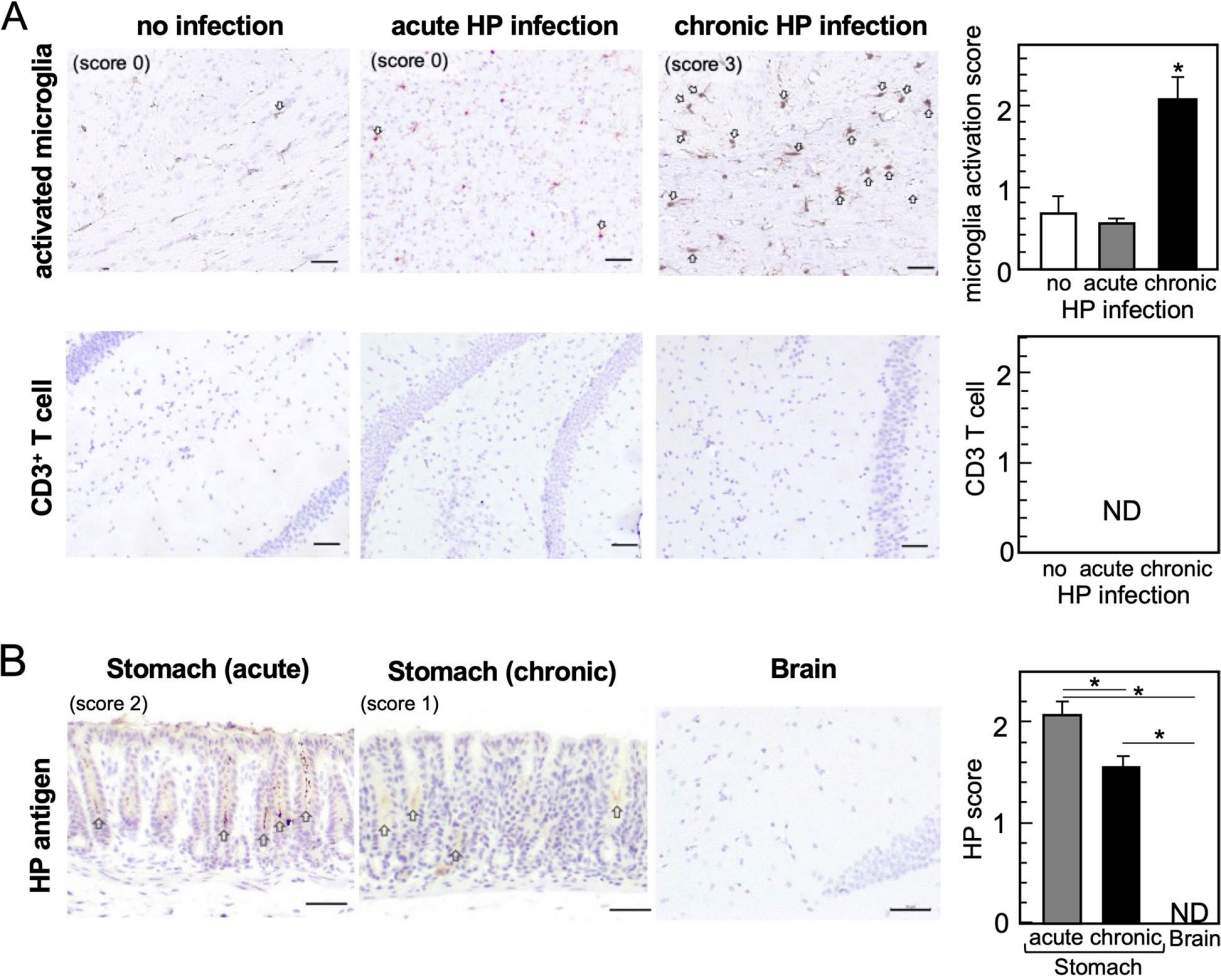
Induction of neuroinflammation by chronic HP infection. Using an oral gavage, we inoculated C57BL/6 mice with 300-μl Brucella broth containing 4 × 10^7^ *H. pylori* daily for 5 days. Three weeks (acute) or 5 months (chronic) after HP infection, we harvested the brain and stomach and conducted immunohistochemistry (IHC) using formalin-fixed and paraffin-embedded sections. **A** Activated microglia (brown color, arrows) and T cells were visualized by antibodies against Iba1 (GeneTex, Irvine, CA) and CD3 (Biocare medical, Concord, CA), respectively. We determined the level of neuroinflammation by the microglia activation score, using five coronal brain slices per mouse; we quantified Iba1-positive cells (positive cells/field 0.1 mm^2^: score 0 = 0–2; 1 = 3–5; 2 = 6–10; 3 = 11–15; 4 = 16–20; and 5 = 21 or more) and calculated the average [mean + standard error of the mean (SEM)] of the acute or chronic HP-infected group and the uninfected control group (nine mice per group). We found that the chronic HP-infected group had significantly higher scores than the control and acute groups. *P < 0.05 by the Student’s *t* test. Bar = 50 μm. We did not detect infiltration of CD3^+^ T cells in the brain parenchyma in any groups. Bar = 50 μm. ND, not detected. **B** IHC of HP antigens in the stomach (pylorus) and brain from HP-infected mice. Using anti-HP antibody (Thermo Fisher Scientific, Waltham, MA), we found HP antigen-positive bacteria (brown color, arrows) in the stomach during the acute and chronic phases, but not in the brain. The HP scores in the stomach were determined as follows: HP antigen-positive gastric crypts/total gastric crypts × 100 (%) (score 0 = negative; 1 = 1–20%; 2 = 21–50%; and 3 = 51–100%). The representative stomach image is scored as 3, which was determined as follow: eight HP-antigen positive crypts/11 total crypts × 100 = 73%. Bar = 50 μm. *P < 0.05 by the Student’s *t* test. ND, not detected

To determine the relationship, we inoculated wild-type (WT) C57BL/6 mice with HP Sydney strain 1 (HP-SS1, belongs to Europe strain, CagA^+^, VacA^+^) and harvested the brain and stomach during the acute [3 weeks post-infection (p.i.)] or chronic (5 months p.i.) phases of HP infection. We examined whether oral HP infection could induce neuroinflammation and HP colonization by immunohistochemistry (IHC). In the brain, we visualized and semi-quantified microglia activation, which are CNS resident innate immune cells, using antibody against ionized calcium-binding adapter protein 1 (Iba1). We found significant activation of microglia in the brain parenchyma during the chronic phase, but not the acute phase, of HP infection (Fig. 2A). On the other hand, we did not observe the extravasation of blood-derived immune cells, including CD3^+^ T cells, in the brain (Fig. 2A). We also detected HP antigens in the stomach, but not in the brain, of all HP-infected mice (Fig. 2B).

Next, we investigated whether HP infection could induce/modulate various immune responses (Fig. 1C, II–IV). We examined the presence of antibodies that react to autoantigens by incubating the control mouse brain sections with HP-infected mouse serum as a primary antibody of IHC. Since we did not detect any positive staining, HP-infection unlikely induced the production of CNS-reactive autoantibodies (data not shown). Using enzyme-linked immunosorbent assays (ELISAs), we quantified serum IgG isotype levels as an indicator of helper T (Th) 1/Th2 balance [27]. We did not see any changes in the levels of IgG isotypes before and after HP infection (data not shown), although the results may reflect the genetic background of mouse strain, where we used C57BL/6 mice with Th1-dominant immune responses [28]. We also quantified the amounts of the pro-inflammatory cytokine IL-6 in serum by ELISAs and found that HP infection did not alter IL-6 production (data not shown).

Since the microglial activation observed in HP infection was not accompanied with the changes in the peripheral immune response, it was reasonable to explore another possibility that HP infection activates microglia in the brain directly by HP components, not via reflection of systemic activation of immune responses. HP infection has been shown to cause the “leaky gut” by disrupting the tight-junctional proteins occludin, claudin-4, and claudin-5 [29]; through the “leaky gut”, the HP components, but not a whole bacterial cell, may penetrate the lamina propria and enter the bloodstream, resulting in subsequent delivery of HP components to the brain. As a candidate of HP components, we introduce the outer membrane vesicles (OMVs).

### Bacterial OMVs and the presence of HP-OMVs in HP-infected mouse sera

**Fig. 3:**
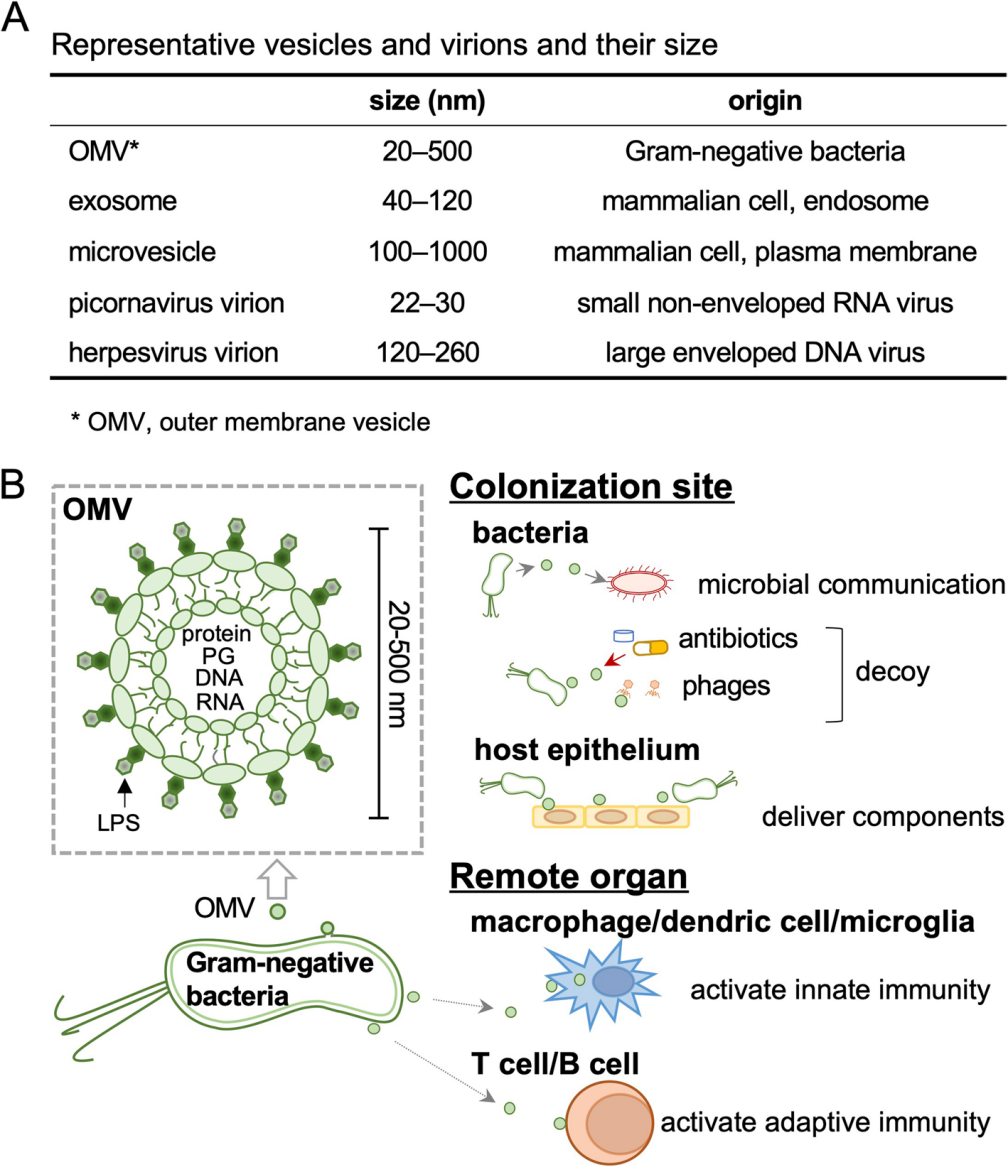
Bacterial outer membrane vesicles (OMVs) and their functions. **A** The size of representative vesicles and virions. Exosomes and microvesicles are produced by mammalian cells. **B** OMVs are derived from the outer membrane lipid bilayer of Gram-negative bacteria; OMVs contain lipopolysaccharides (LPS), proteins, peptidoglycans (PG), DNA, and RNA. OMVs have various functions. At the colonization site, OMVs support the communications between bacteria, serve as a decoy target for antibiotics and bacteriophages, and transfer virulence factors, such as toxins, to target host cells (epithelium). In the remote organs, OMVs can activate innate (macrophage/dendritic cell/microglia) and adaptive (T cell/B cell) immune cells

Most Gram-negative bacteria release OMVs into their environment [30, 31]. OMVs are 20–500 nm in diameter, composed of the lipid bilayer derived from the outer membrane of bacteria, and contain bacterial components, including lipopolysaccharides (LPS), proteins, peptidoglycans, DNA, and RNA (Fig. 3). Mammalian cells also produce extracellular vesicles (EVs), named exosomes and microvesicles. Exosomes and microvesicles originate from endosomes and plasma membranes, respectively, and play roles in intercellular communications [32]. The size of OMVs is similar to that of EVs and viral particles (virions). Figure 3A shows the size of representative small and large viruses, i.e., picornavirus and herpesvirus, respectively. A variety of viruses that belong to different families are known to enter the CNS (“neuroinvasive” viruses) via axonal transport, regardless of the virion size.

Functionally, at the colonization sites, OMVs are implicated in inter-microbial communications through exchanges of genetic materials, chemical compounds, and signaling molecules [33, 34] (Fig. 3B). The communications can be conducted between the same and different bacterial strains [32]. OMVs may also play direct roles in bacterial survival [35, 36], since OMVs can increase the resistance to antibiotics and bacteriophages by serving as decoy targets of these molecules [33]. Furthermore, OMVs can be used for the delivery of bacterial virulence factors, such as toxins, into host epithelial cells at the colonization sites. On the other hand, OMVs can activate both innate (macrophage/dendritic cell/microglia) and adaptive (T cell/B cell) immune cells in the remote organs once OMVs are delivered, since the components of OMVs, such as LPS, are known as activators of various immune response pathways [34].

Clinically, there are a limited number of reports that OMVs were detected in human body fluids. For example, OMVs have been detected in blood of patients with sepsis [37] and inflammatory bowel disease [38]. Although OMV production has been confirmed in cultured broth and the functional analyses have been performed mainly in vitro [33, 39–41], only a few experimental studies have been reported on the detection of OMVs in the general organs and systemic circulation, or their in vivo functions. This may be due to the technical difficulty to isolate bacterial OMVs versus host cell-derived EVs separately from body fluid because of their molecule-size similarities, although a protocol for the separate isolation of OMVs and EVs has been published recently [42].

**Fig. 4:**
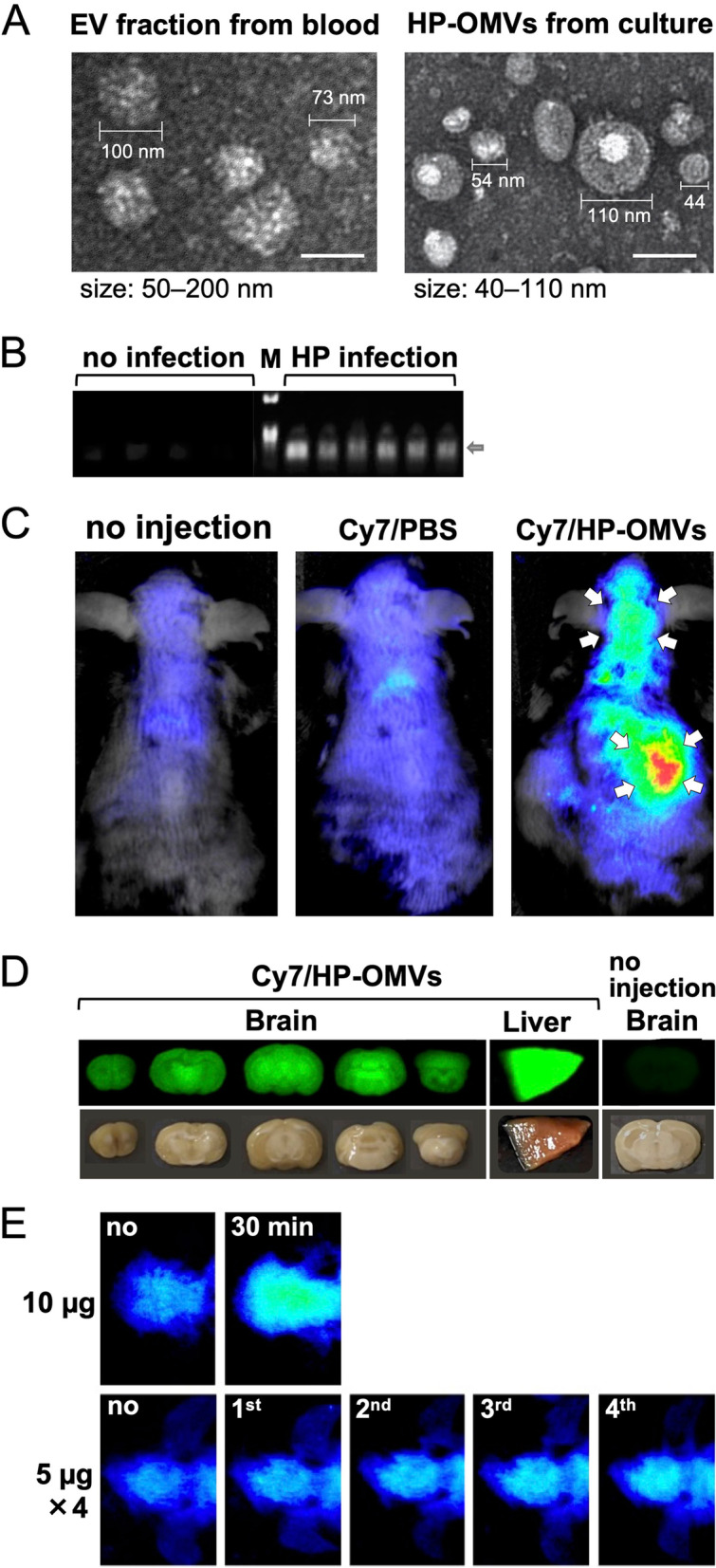
Isolation of HP-OMVs and their distribution in the body. **A** Electron microscopic images of the extracellular vesicle (EV) fraction from HP-infected mouse sera and HP-OMVs. Five months after HP infection, we collected the EV fractions from sera, using a total exosome isolation kit. We prepared HP-OMVs from an HP-SS1-cultured broth by filtration and ultracentrifugation. The size of vesicles from the EV fractions and HP-OMVs were 50–200 nm and 40–110 nm, respectively. Bar = 100 nm. **B** EV fractions were collected from HP-infected and uninfected mouse sera. A HP-specific 16S rRNA gene in the EV fractions was amplified by PCR and the products were subjected to agarose gel electrophoresis (arrow). M, DNA size marker. The HP-specific 16S rRNA gene (arrow) was detected only in the EV fractions from HP-infected mice but not in uninfected mice. **C**–**E** We isolated HP-OMVs from HP-cultured broth and labeled them with Hilyte Fluor 750 (Cy7) labeling Kit-NH2 (Dojindo Laboratory, Kumamoto, Japan). Cy7-labeled HP-OMVs (Cy7/HP-OMV, 10 μg/mouse) or control Cy7-containing phosphate-buffered saline (Cy7/PBS) was injected into mice intravenously, and the fluorescence was monitored in live mice by the Pearl Trilogy Imaging System (SCRUM, Inc., Tokyo, Japan). **C** Representative images of the dorsal side at 30 min after injection. The mouse injected with 10 μg of Cy7/HP-OMVs had a high fluorescent intensity around the liver and head areas (arrows) (*n* = 7). **D** 40 min after injection of Cy7/HP-OMVs, we killed and perfused mice with PBS, prepared the coronal brain sections, and examined the fluorescent intensities. We used the liver from the Cy7/HP-OMVs-injected mice as a positive control; we used the brain from non-injected mice as a negative control, confirming no autofluorescence in the brain of naïve mice. **E** We compared the Cy7 intensities of the head area between the mice receiving the 10 μg of a Cy7/HP-OMV single injection and mice receiving four injections of the 5 μg of Cy7/HP-OMVs with 30 min intervals (*n* = 7 and 2, respectively). Images of 4-time injections were taken 25 min after each injection; no, image was taken at 0 min; 1st, first injection at 0 min and the image was taken at 25 min; 2nd, the second injection at 30 min and the image was taken at 55 min; 3rd, the third injection at 60 min and the image was taken at 85 min; and 4th, the fourth injection at 90 min and the image was taken at 115 min. The fluorescent intensities increased depending on the number of injections

HP-derived OMVs (HP-OMVs) were detected in gastric fluids of patients with gastric cancer [41]. In contrast, there are no reports demonstrating the presence of HP-OMVs in blood, although the presence of HP DNA has been detected in cell-free DNA (cfDNA) from plasma [43]; cfDNA extracted from liquid biopsy samples, such as plasma, has been shown to be useful for the diagnosis of infections [44]. Thus, we investigated whether HP-OMVs could be detectable in blood from chronically HP-infected mice. We harvested sera from the mice 5 months p.i., centrifuged sera at 10,000×*g* for 20 min, and collected the supernatants; in this step, host cells were removed as the pellets (of note: any potential contaminated bacterial cells should also be removed in this step, although there was no HP bacteremia in our experimental condition). Then, we collected the “EV fraction” from the supernatants using a total exosome isolation kit (Invitrogen, Waltham, MA). The EV fraction contained both host’s EVs (exosomes and microvesicles) and bacterial OMVs, since the isolation kit is designed to collect any hydrophobic vesicles. For comparisons and further experiments, we also prepared HP-OMVs from HP-SS1-cultured broth (Brucella broth containing 7% fetal bovine serum) by a standard method [40]. Using the electron microscopic images (Hitachi, Tokyo, Japan), we determined the size of vesicles in the serum EV fractions and HP-OMVs as 50–200 nm and 40–110 nm, respectively (Fig. 4A). We used the EV fractions to perform PCR with an HP-specific 16S rRNA primer set. We found that the EV fractions from the HP-infected mice were positive for HP-specific DNA (Fig. 4B), suggesting that HP-OMVs were present in blood of HP-infected mice.

### Potential OMV delivery to the brain

Although gut-derived OMVs can enter blood as described above, OMVs need to cross the blood-brain barrier (BBB) to enter the brain. The BBB is composed of astrocyte foot processes and specialized endothelial cells that lack pores, have few pinocytic vesicles to minimize uptake of extracellular substances, and have extensive tight junctions that severely restrict cell permeability. Limited permeability restricts the movement of substances from the systemic circulation to the CNS, thereby protecting the CNS from exposure to molecules that may be toxic to neurons. Under the normal physiological conditions, since only the hydrophobic molecules with less than 400 daltons can pass through the BBB, the transportation of most molecules into the CNS is blocked by the BBB. On the other hand, under certain conditions, such as inflammation or ischemic stroke, the BBB breakdown occurs and allows the passage of larger and hydrophilic substances. Intestinal dysbiosis has also been proposed to cause the “leaky” BBB. A germ-free mouse had weaker BBB functions with lower levels of tight junction proteins, compared with the control; tight junction proteins were increased by short-chain fatty acids (SCFAs), such as propionate and acetate, which are produced by the gut microbiota [45]. On the other hand, some components of OMVs may contribute to the “leaky” BBB. LPS caused the “leaky” BBB [30, 46] by reducing the number of tight junction proteins, ZO-1 and claudin-5, as well as by disrupting the intercellular adhesion molecules, such as cadherin [47–50]. OMVs from the periodontal pathogen *Porphyromonas gingivalis* contain cysteine proteinases, named gingipains, which degrade tight junction proteins and increase BBB permeability in vitro [49, 50].

Two in vivo studies showed the presence of OMVs in the brain following peripheral injection of OMVs, suggesting that OMVs can cross the BBB. If this is the case, OMVs can be a far-reaching horizontal shuttle system from the gut lumen to the CNS, serving as a communication tool to maintain and modulate microbe-host interactions. Lee et al. demonstrated the entry of exogenously injected OMVs to the brain, using a mouse model [51, 52]. They prepared OMVs from the periodontal pathogen *Aggregatibacter actinomycetemcomitans* and injected the OMVs into mice intracardially or intravenously. After 24 h of injection, they detected the presence of OMVs and an increase of TNF-α in the brain [47]. They also showed the uptake of OMVs by meningeal macrophages and microglia [48]. Bittel et al. demonstrated the transfer of gut bacteria-derived OMVs to a wide range of host organs, including the brain, of experimental mice [53]. The authors visualized *Escherichia coli-*derived OMVs (EC-OMVs) in the body, using the Cre-loxP system; Cre-expressing-EC was prepared and administered orally to the mice, which can produce red fluorescence protein in the presence of Cre protein. This system enabled the visualization of EC-OMV entry to the mouse cytoplasm by detecting red fluorescent proteins.

We investigated the potential tissue distribution of HP-OMVs by injecting mice with fluorescence cyanine 7 (Cy7)-labeled HP-OMVs (Cy7/HP-OMVs). We intravenously injected 10 μg of Cy7/HP-OMVs or control Cy7-containing phosphate-buffered saline (Cy7/PBS) into 2-year-old WT mice from the tail vein. The Cy7 fluorescence was monitored in live mice by Pearl Trilogy Imaging System (SCRUM, Inc., Tokyo, Japan). We detected high Cy7 fluorescent intensities around the liver and head areas in mice injected with Cy7/HP-OMVs, but not in the control mice injected with Cy7/PBS (Fig. 4C). Then, we killed mice and prepared the coronal brain sections 40 min after the Cy7/HP-OMV injection to examine whether we could detect the Cy7 fluorescence in the brain parenchyma (Fig. 4D). We found the fluorescent intensities in the brain parenchyma of all five coronal sections; strong fluorescent intensities were observed in the gray matter, including the caudoputamen, the thalamus, and the granular layer of the cerebellum. The above experiments were conducted using a high dose of fluorescent OMVs (10 μg per injection); then, we tested whether a lower dose [54] of Cy7/HP-OMVs (5 μg per injection) could also result in the accumulation of Cy7/HP-OMVs in the brain by injecting four times with 30-min intervals (Fig. 4E). We found that the Cy7 intensities became more detectable dependent on the number of injections of the 5 μg of Cy7/HP-OMVs. However, the intensities were weaker than the Cy7 intensity following the 10 μg of Cy7/HP-OMV injection, suggesting that the clearance of OMVs seemed to depend on the concentrations.

### Pro-inflammatory effects of HP-OMVs on immune cells and neuronal cells

Jarzab et al. [55] reviewed the characteristics of HP-OMVs. The size (20–500 nm) and amount of HP-OMVs varied depending on the HP strain, growth phase, and environment. The proteins contained in OMVs were not only the surface proteins but also the virulence factors, such as urease, CagA, and VacA [39, 56]. The pro-inflammatory effects of HP-OMVs on gastrointestinal and immune cells have been demonstrated by in vitro experiments. On the human gastric epithelial cell line AGS and colonic epithelial cell line T84, HP-OMVs induced secretion of IL-8, leading to chemotaxis toward neutrophils and other granulocytes as well as phagocytosis [39, 40]. On isolated human peripheral blood mononuclear cells, HP-OMVs stimulated the release of pro-inflammatory cytokine IL-6 and anti-inflammatory cytokine IL-10. Choi et al. demonstrated that HP-OMVs enhanced TNF-α and IL-6 production from mouse macrophage cell line RAW 264.7 in vitro [41]. The authors also found that following in vitro stimulation with anti-CD3/CD28 antibodies, splenic cells from in vivo HP-OMVs-injected mice had larger amounts of interferon-γ and IL-17 than those from the control mice [41].

**Fig. 5:**
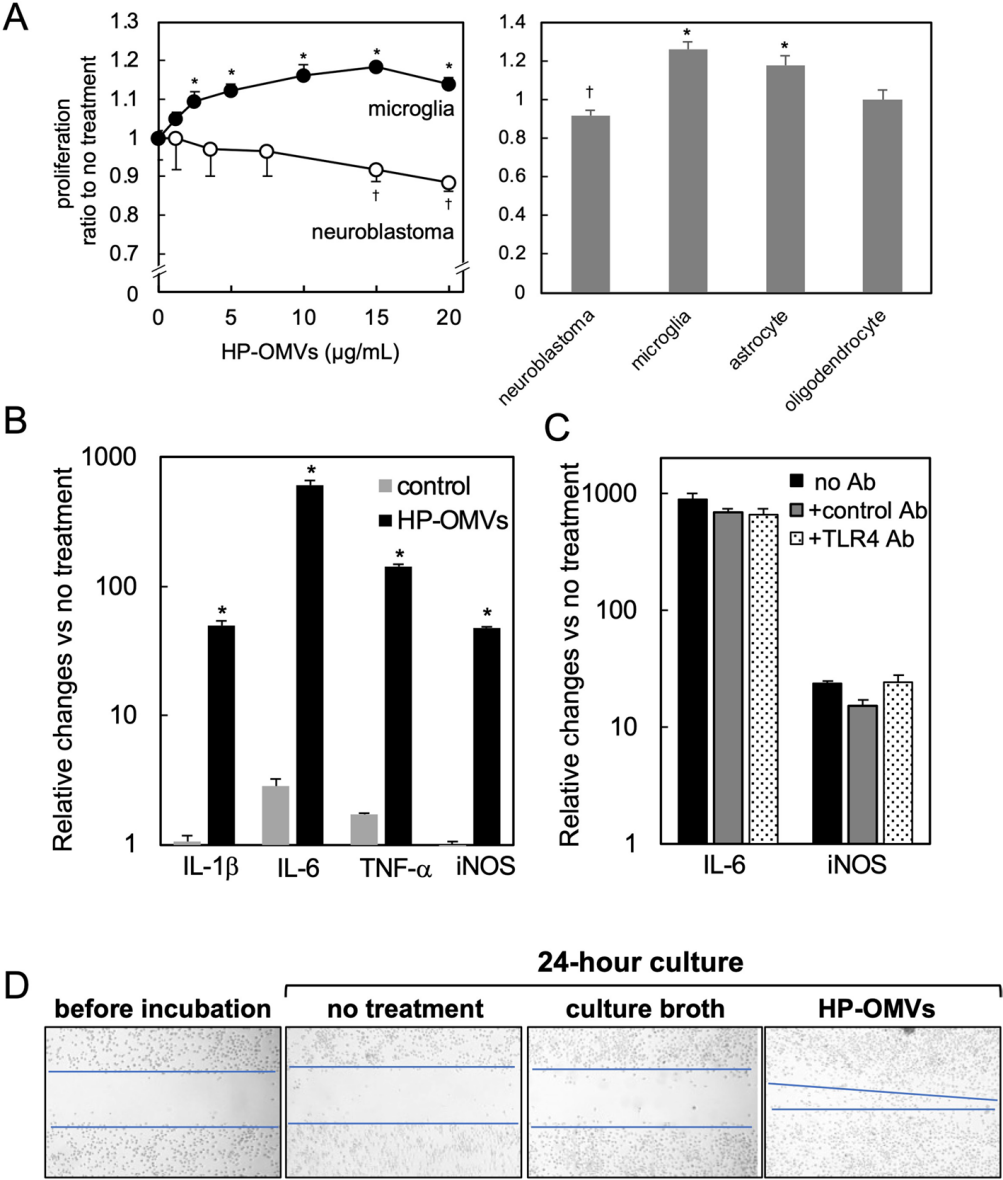
Enhancement of microglial proliferation, cytokine production, and migration. We determined the effects of HP-OMVs on cell proliferation, using four neuronal and glial cell lines (neuroblastoma, microglia, astrocyte, and oligodendrocyte). **A** (Left panel) HP-OMVs enhanced microglia proliferation but suppressed neuroblastoma proliferation. We incubated the cells with HP-OMVs for 24 h with different concentrations of HP-OMVs, then added the CCK-8 reagent for 1 h and measured the optical density at 450 nm. We calculated the proliferation ratio, dividing the optical density of the samples by that of untreated samples. **A** (Right panel) Four neuronal cell lines were incubated with 15 μg/mL of HP-OMVs for 24 h, and proliferation was determined by CCK-8 assay. HP-OMVs also enhanced proliferation of astrocytes, but not oligodendrocytes. †, significant suppression (*P* < 0.05, Student’s *t* test) compared with untreated control neuroblastoma; *, significant enhancement (*P* < 0.05) compared with untreated microglia or astrocytes. **B** We treated microglia with HP-OMVs for 9 h and determined the relative changes of mRNA levels of pro-inflammatory molecules (HP-OMV, black column), compared with the no treatment group, by real-time quantitative PCR (qPCR). HP-OMVs significantly upregulated the levels of interleukin (IL)-1β, IL-6, tumor necrosis factor (TNF)-α, and inducible nitric oxide synthase (iNOS). **C** Following 1-h pretreatment with toll-like receptor 4 (TLR4)-neutralizing antibody (Ab)(TLR4 Ab, hatched column) or the control Ab (gray column), we incubated microglia with HP-OMVs for 9 h. We found no differences in the mRNA levels of IL-6 or iNOS, compared with the group incubated with HP-OMVs alone (no Ab, black column). **D** We determined microglial migrative activities using a scratch assay with or without HP-OMV incubation. The microglial monolayer was scraped into a straight line to create a wound and cultured for 24 h. We took wound-closure images and plotted the interfaces (blue line) between the wound and cell fronts. The wound size was reduced only in HP-OMV culture, but not in two control cultures (no treatment and culture broth, see text). In all experiments in Fig. 5, *n* = 3 per group, and the experiments were repeated at least twice. *P < 0.05, compared with the controls by ANOVA

In the brain parenchyma, there are four types of cells: neuron, microglia, astrocyte, and oligodendrocyte. Among them, microglia have been used for determining the effects of OMVs. For example, Ha et al. stimulated microglial cell lines with OMVs derived from *A. actinomycetemcomitans* and found increased levels of IL-6 secretion and mRNA expression [52]. On the other hand, there has been few reports demonstrating the effects of OMVs on other brain cells. Thus, we examined the effect of HP-OMVs on the proliferation of each neuronal or glial cell line: neuroblastoma (Neuro-2a, ATCC# CCL-131), microglia (MG6) [57, 58], astrocyte [59], and oligodendrocyte (OS3) [60] (Fig. 5A). Using a similar range of OMV concentrations published previously [40, 61], we incubated neuroblastoma and microglia with HP-OMVs for 24 h and determined the levels of cell proliferation using the Cell Counting Kit-8 (CCK8, Dojindo Laboratory). We found that HP-OMVs dose-dependently suppressed the proliferation of neuroblastoma and enhanced that of microglia (Fig. 5A). When we incubated the four cell lines with HP-OMVs (15 μg/mL), HP-OMVs significantly enhanced the proliferation of astrocytes, and had no effects on oligodendrocyte proliferation (Fig. 5A).

Since we found microglia activation in HP-infected mice (Fig. 2), we further investigated the effect of HP-OMVs on microglia. We examined mRNA levels of pro-inflammatory cytokines (IL-1β, IL-6, and TNF-α) and inducible nitric oxide synthase (iNOS) in microglial cultures after 9-h incubation with HP-OMVs. Using real-time quantitative PCR, we found that HP-OMV incubation significantly upregulated all the mRNA levels, compared with the controls (Fig. 5B). In the control cultures, we incubated the cells with a culture broth pellet suspension prepared by the same procedure of HP-OMV preparation [40]. Among components of OMVs, LPS is a candidate molecule that can induce the pro-inflammatory molecule production [62]. Since LPS is a ligand of toll-like receptor (TLR) 4, we pretreated microglia with TLR4-neutralizing antibodies and then incubated the microglia with HP-OMVs. We found that TLR4-neutralizing antibody treatment did not suppress the expression levels of IL-6 or iNOS gene in microglia incubated with HP-OMVs (Fig. 5C), suggesting that microglial activation by HP-OMVs can be caused by other molecules rather than LPS.

Microglial migration can be facilitated by several molecules: peptides and proteins (cytokines and chemokines), small hydrophilic molecules (nucleotides), and bioactive lipids. Among bacterial components, gingipain, but not LPS, induced microglial migration [56, 57]. Thus, we examined whether HP-OMVs could induce microglial migration by the scratch assays. We created a wound by scraping the microglial monolayer in a straight line and cultured microglia in the presence or absence of HP-OMVs for 24 h. To determine the effects of HP-OMVs on microglial migration, we took wound closure images using a microscope (Olympus, Tokyo, Japan). We found that HP-OMVs induced microglia migration (Fig. 5D), although we found no migration in the two control cultures: no treatment and the control (incubated with a culture broth pellet suspension). Microglia migration can be triggered by the activation of intracellular signals, such as mitogen-activated protein kinase (MAPK) [56]; this is consistent with our finding that HP-OMV incubation activated p38 MAPK (data not shown).

### AD pathology, bacterial OMVs, and HP infection

**Fig. 6:**
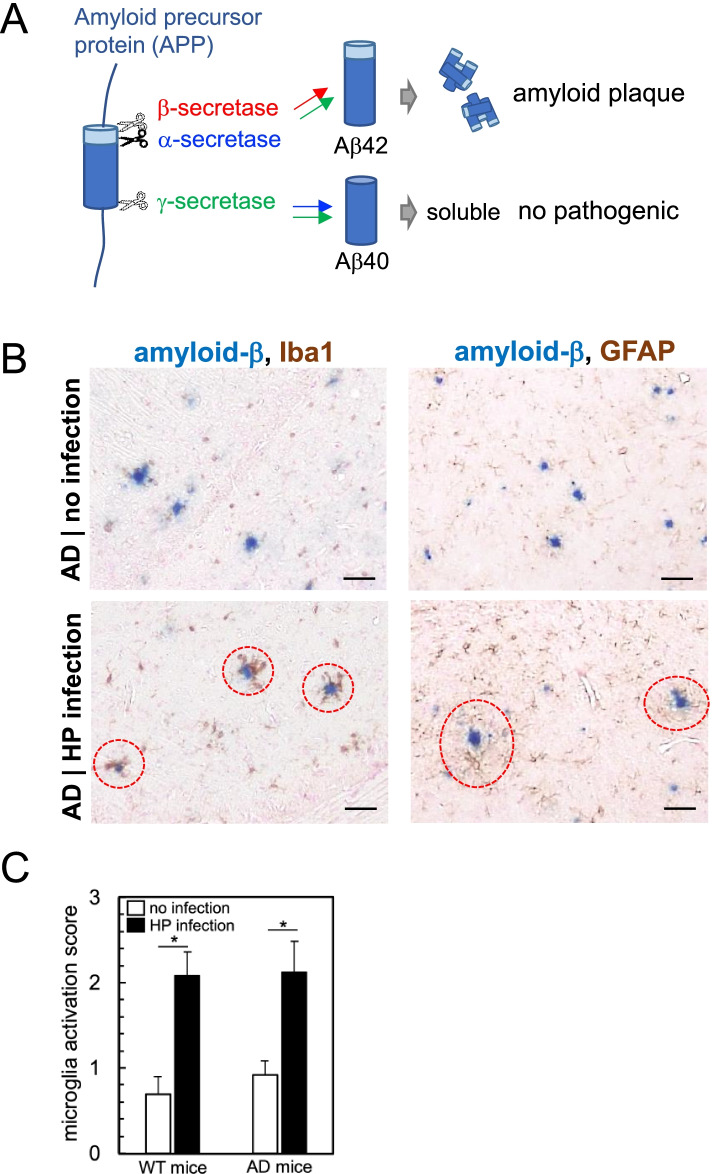
Co-localization of amyloid-β (Aβ) and activated glial cells in HP-infected mouse brain. **A** Amyloid precursor protein (APP) is cleaved by three types of secretases. APP cleavage by β-secretase/γ-secretase produces the plaque-forming pathogenic Aβ42 (42 amino acids in length). APP cleavage by α-secretase/γ-secretase produces the soluble Aβ40 (40 amino acids in length). **B** AD model mice (double transgenic of mutant APP and mutant PS1) were infected with HP. Five months later, we harvested the brain and compared the brain sections with that of uninfected AD model mice. Representative IHC double-staining images of Aβ (blue color) and activated microglia (Iba1, brown color, left panels) or astrocytes (GFAP, brown color, right panels) are shown. In HP-infected AD model mice, Aβ plaques (red dotted circles) were observed adjacent to activated microglia (left) and astrocytes (right). Anti-Aβ and GFAP antibodies were purchased from Cell Signaling (Beverly, MA). **C** Microglial activation scores were compared between HP-infected versus uninfected mice. *n* = 9/group, *P < 0.05, compared with uninfected mice (Student’s *t* test)

Dementia involves deterioration in cognitive functions beyond what is expected from usual biological aging. Currently, more than 55 million people live with dementia worldwide, and there are nearly 10 million new cases every year. AD is the most common form of dementia, contributing to 60–70% of dementia cases [63]. AD is characterized by the aggregation and accumulation of amyloid-β in the brain parenchyma and hyperphosphorylated tau in neurons as neurofibrillary tangles. Amyloid-β is produced by cleavage of amyloid precursor protein (APP) (Fig. 6A); APP is cleaved by β-secretase and γ-secretase to generate the pathogenic plaque-forming amyloid-β, Aβ42 (42 amino acids in length). When APP is cleaved by α-secretase and γ-secretase, the soluble non-pathogenic amyloid-β, Aβ40 (40 amino acids in length) is generated. The γ-secretase complex is composed of four proteins: presenilin (PS), nicastrin (NCT), anterior pharynx-defective 1 (APH-1), and presenilin enhancer 2 (PEN2); either PS1 or PS2 is included in the γ-secretase complex. PS1 and PS2 belong to the PS family; PS gene mutations have been linked to the early onset of AD [64]. In AD, chronic neuroinflammation also contributes to the progression of neuropathological changes [24]. There are two types of neuroinflammation. One is caused by activation of resident innate immune cells (microglia and astrocytes) and the other is characterized by infiltration of blood-derived immune components, including T cells and antibodies, into the CNS. Neuroinflammation in AD is the former type. Activated microglia produce inflammatory and toxic products, which are involved in the amyloid-β generation and tangle formation [24].

Risk factors of AD include older age, the female gender, family history [65], and diseases, such as diabetes, hypertension, and stroke. AD development has been linked to infections with various microbes, including herpesvirus [40, 66], periodontal pathogens, especially *A. actinomycetemcomitans* and *P. gingivalis* [67, 68]; HP infection has also been positively associated with AD [19, 23, 24] in epidemiological studies. In Table 1, we summarized the published epidemiological data on the HP prevalence, HP strain, the prevalence of AD and other dementia, and the risk ratio of HP infection to AD in the world, including Japan, China, the US, four European countries, Turkey, and Brazil. Overall, the countries with a high HP prevalence, particularly Turkey and Brazil, had a high prevalence of AD and other dementias. On the other hand, the countries with a low HP prevalence, such as the USA and Sweden, had a low prevalence of dementias. Based on the risk ratio, AD development seemed independent of the HP strains. Since each HP strain has differences in major virulence factors CagA and VacA [4], these data imply that CagA and VacA likely play no role in AD development or neuroinflammation, although CagA and VacA are pathogenic in gastric diseases. In clinical research, it is difficult to demonstrate a direct link between HP infection and AD because of the great variation in patients’ lifestyles and medical history, especially among patients of advanced age. Thus, we investigated the roles of HP infection in a mouse model of AD. We used AD model mice, which were double transgenic mice expressing a chimeric mouse/human APP (Mo/HuAPP695swe) and a mutant human PS1 (PS1-dE9) with a C57BL/6 genetic background. Both proteins are expressed in CNS neurons and cause amyloid-β accumulation. Five months after HP infection, we harvested the brain and conducted double immunostaining to determine the co-localization of amyloid-β and activated microglia or astrocytes. The activated microglia and astrocytes were observed adjacent to amyloid-β plaques in HP-infected AD model mice, but not in uninfected AD model mice (Fig. 6B). Although the levels of activated microglia were significantly increased in HP-infected AD model mice, compared with uninfected AD model mice, the levels of activated microglia were similar between HP-infected AD model mice and HP-infected WT mice (Fig. 2A, Fig. 6C). On the other hand, the amyloid-β plaque levels were unchanged by HP infection (data not shown).

**Table 1:** Epidemiological data on HP infection and its association with AD

	Japan	China	USA	France	Sweden	Netherland	Greece	Turkey	Brazil	Ref
HP infection (%)	50–60	70–80	20–30	20–30	< 20	40–50	40–50	80–90	80–90	[69]
HP strain	East Asia	East Asia	Europe	Europe	Europe	Europe	Europe	Europe	Europe/Africa	[6]
AD and other dementias (%)	0.8–0.9	0.8–0.9	0.5–0.6	0.6–0.7	0.5–0.6	0.5–0.6	0.6–0.7	1.1–1.2	1–1.1	[65]
Risk ratio of HP infection to AD	0.94	0.66/1.39	1.45	2.85	1.21	1.06	2.76/8.4	ND	ND	[23]

*Abbreviation*s: *AD,* Alzheimer’s disease; *HP*, *Helicobacter pylori*; *ND,* not determined

In summary, the in vivo experiments showed that HP infection activated microglia, which co-localized amyloid-β plaques. This is consistent with in vitro data in which HP-OMVs enhanced microglial proliferation, cytokine production, and migration. In contrast, increased levels of amyloid-β-producing enzymes by HP-OMV incubation in vitro were inconsistent with unchanged amyloid-β levels in HP-infected mouse brains. A longer observation period may be required to see the statistical differences in amyloid plaque formation in vivo. Overall, however, our current results showed the association between chronic HP infection and AD pathology.

## Conclusion: a working hypothesis

Recently, Wei et al. proposed a potential harmful role of OMVs in human AD, based on the experiments of intravenous OMV transfer into mice, in which OMVs were derived from the feces of healthy controls or those of patients with AD [70]. The mice receiving AD-derived OMVs had the “leaky” BBB and worse memory test results with activated taurine phosphorylation and microglial activation in the brain. In this study, it is unclear which bacterial OMVs contributed to the changes in the transferred-mice, since the authors combined 15 fecal samples from patients with AD and did not analyze the fecal microbiota. However, these results suggest that feces-derived OMVs in blood could affect brain functions once OMVs enter the systemic circulation.

**Fig. 7:**
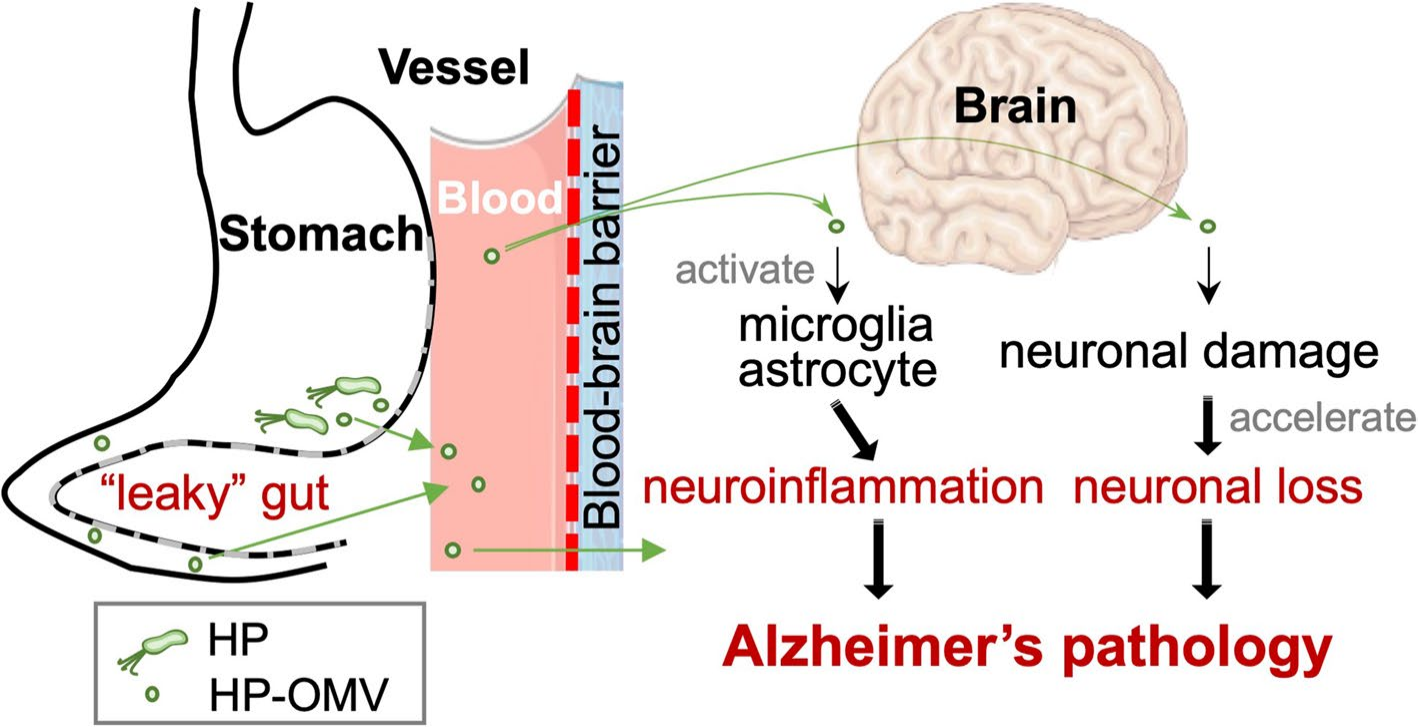
A working hypothesis on the roles of HP-OMVs in Alzheimer’s disease (AD) pathology. HP colonizes in the stomach chronically and produces OMVs that disrupt tight junctions in the gut epithelium. Through the “leaky” gut, HP-OMVs may enter the bloodstream. Next, the circulating HP-OMVs can increase the permeability of the blood-brain barrier (BBB), resulting in BBB breakdown which allows HP-OMVs to enter the brain parenchyma. In the brain, OMVs cause activation and/or migration of microglia and astrocytes as well as enhance neuronal damage initiated by amyloid-β or tau, accelerating neuronal loss. This would lead to neuroinflammation adjacent to increased amyloid plaques, contributing to AD pathology. Events possibly caused by HP are in red

Our current working hypothesis on the role of HP infection in AD pathology is that chronic HP infection causes neuroinflammation by producing OMVs (Fig. 7). In the stomach, HP continuously produces OMVs that contain bacterial components, including virulent factors. Since HP infection causes the “leaky” gut [29, 71], HP-OMVs may enter the bloodstream through the inflamed stomach. Then, blood HP-OMVs can increase the permeability of the BBB (“leaky” BBB), crossing the BBB. Once HP-OMVs enter the brain, HP-OMVs not only induce activation and/or migration of microglia and astrocytes, but also enhance neuronal damage initiated by amyloid-β or tau, accelerating neuronal loss. This would cause AD development and/or exacerbation. To prove this hypothesis, it is crucial to examine the presence of HP-OMVs in the blood and the distribution of HP-OMVs in the CNS in HP-infected humans, as well as to examine whether the HP eradication by antibiotics can suppress neuroinflammation and AD pathology in HP-infected AD model mice. Although HP DNA has been detected in plasma cfDNA samples [43], most AD studies have used whole blood samples, not sera or plasma, to detect bacterial components in the blood, which are inappropriate for detecting OMVs. The cfDNA analyses in liquid biopsies, using plasma/serum, would determine whether the levels of HP-OMVs could be associated with the development of AD or not.

Although the pathogenesis of AD has been extensively studied, there is no effective treatment to reverse AD’s progression. Thus, the prevention of AD development is the most straightforward strategy. Although HP infects for a long time, it can be eradicated by antibiotics. HP diagnosis followed by its eradication may reduce the risk of AD development and neuroinflammation in general.

## Data Availability

The datasets of the current study are available from the corresponding author on reasonable request.

## Abbreviations

AD: Alzheimer’s disease
APP: Amyloid precursor protein
AQP4: Aquaporin 4
BBB: Blood-brain barrier
CagA: Cytotoxin-associated gene A
cfDNA: Cell-free DNA
CNS: Central nervous system
CRP: C-reactive protein
EC: *Escherichia coli*
EV: Extracellular vesicle
GP: Glycoprotein
HP: *Helicobacter pylori*
Iba1: Ionized calcium binding adapter protein 1
IHC: Immunohistochemistry
IL: Interleukin
iNOS: Inducible nitric oxide synthase
ITP: Idiopathic thrombocytopenic purpura
LPS: Lipopolysaccharide
MALT: Mucosa-associated lymphoid tissue
MAPK: Mitogen-activated protein kinase
MS: Multiple sclerosis
NMO: Neuromyelitis optica
OMV: Outer membrane vesicle
PD: Parkinson’s disease
PG: Peptidoglycan
PS: Presenilin
Th: T helper
TLR: Toll-like receptor
TNF: Tumor necrosis factor
Treg: Regulatory T cell
VacA: Vacuolating toxin A
WT: Wild-type
